# Supplementary figures and images for: Uncovering the genetic diversity in Aedes aegypti insecticide resistance genes through global comparative genomics
Source: Sci Rep. 2024 Jun 11;14:13447. doi: 10.1038/s41598-024-64007-6 (PMC11166649; doi:10.1038/s41598-024-64007-6)

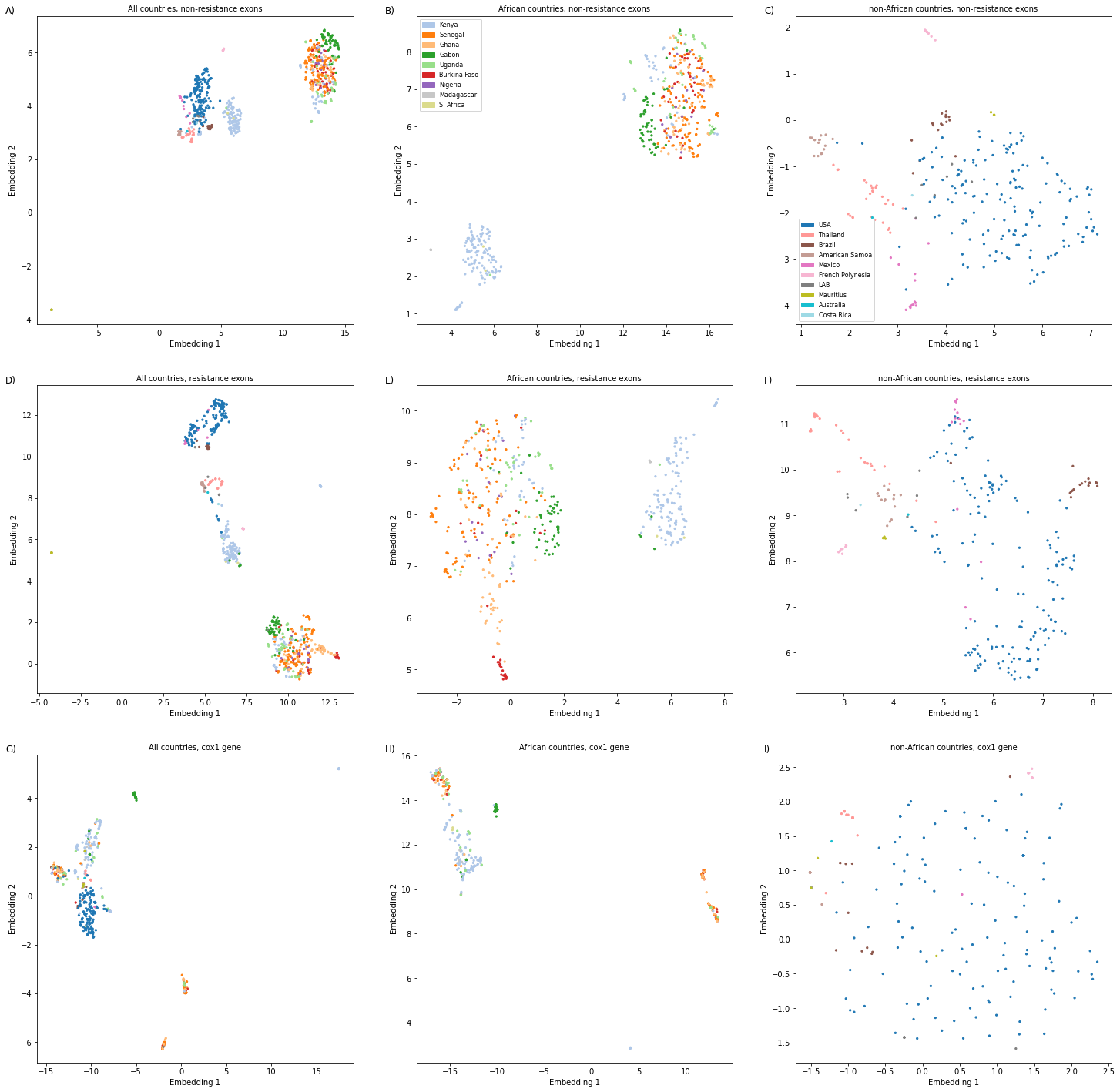

Supplement: Supplementary file 1 — Supplementary Information 1. [file 41598_2024_64007_MOESM1_ESM.png]
