# Supplementary material for: Uncovering the genetic diversity in Aedes aegypti insecticide resistance genes through global comparative genomics
Source: Sci Rep. 2024 Jun 11;14:13447. doi: 10.1038/s41598-024-64007-6 (PMC11166649; doi:10.1038/s41598-024-64007-6)
Supplement: Supplementary file 3 — Supplementary Figures. [file 41598_2024_64007_MOESM3_ESM.docx]

# **SUPPLEMENTARY FIGURES AND TABLES**


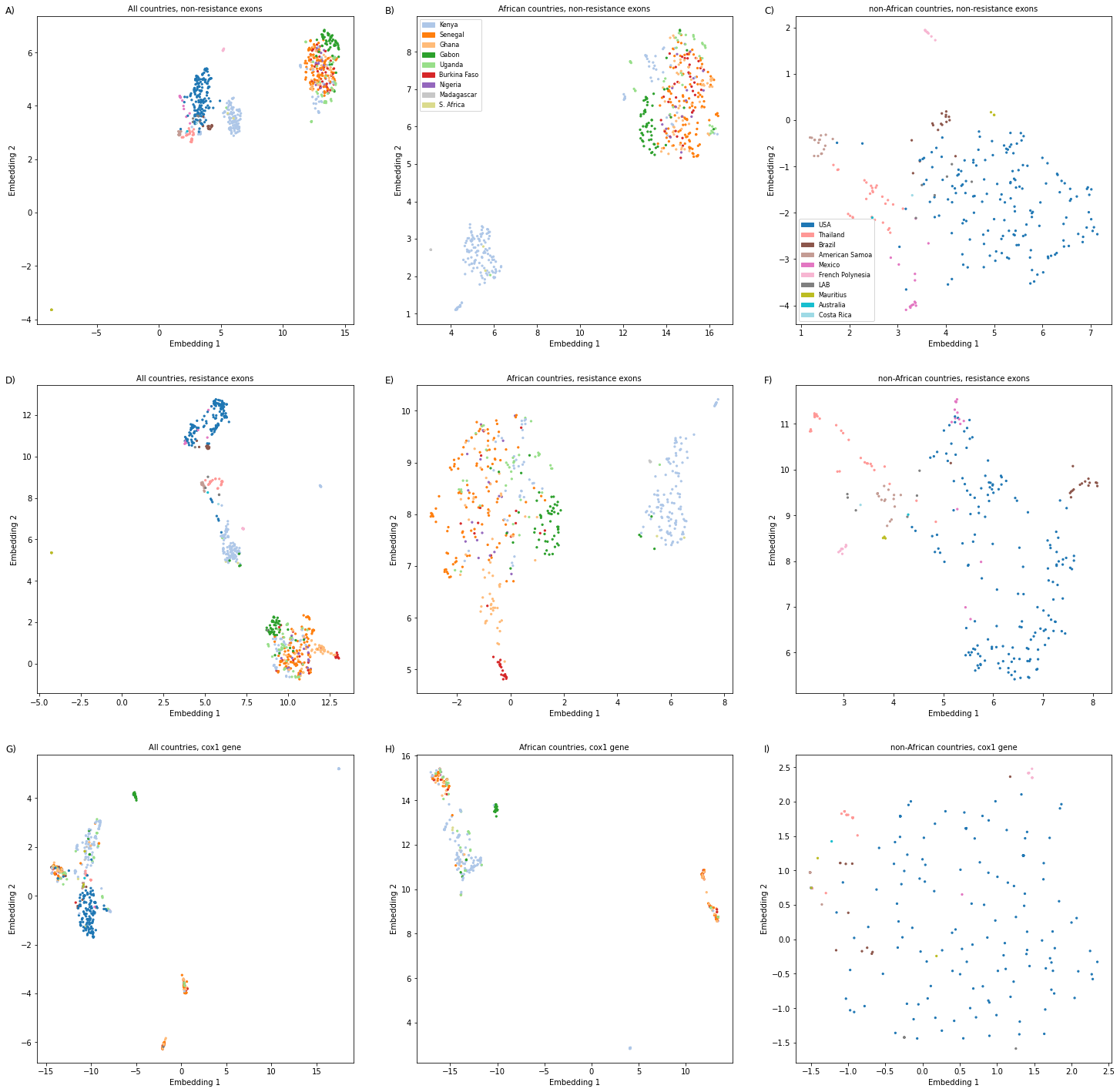


**Supplementary Figure 1. Population structure using UMAP embedding of SNPs for different geographical regions.**


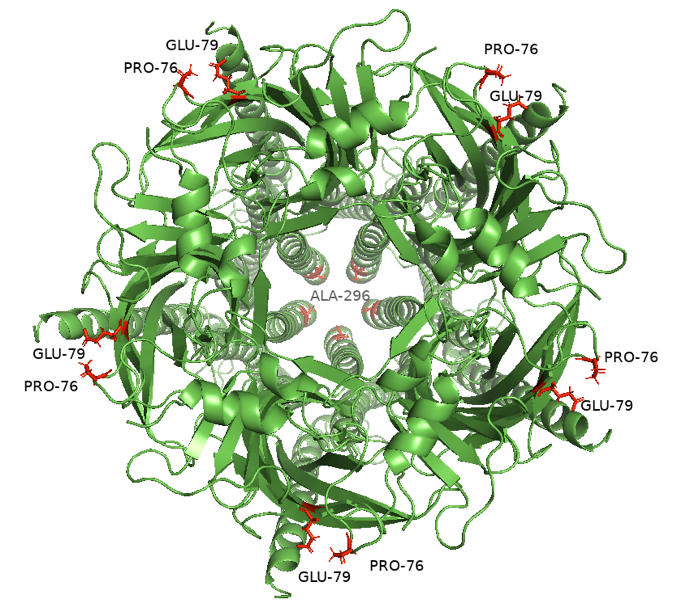


**Supplementary Figure 2. GABA receptor protein structure including mutations found in >10 isolates.**

A. B.


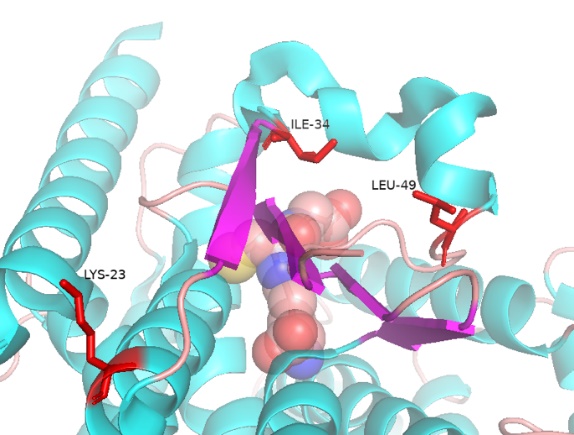

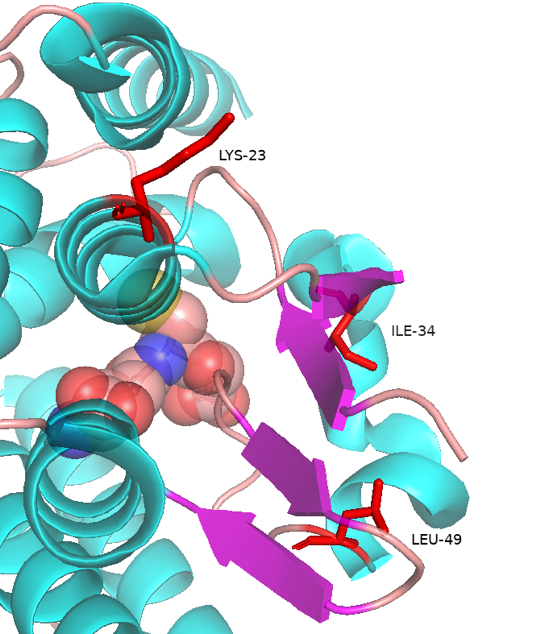


C.
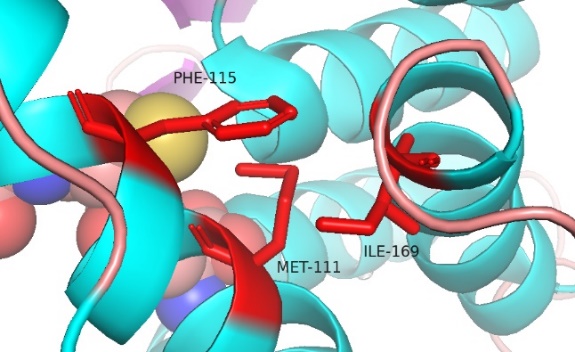


**Supplementary Figure 3. *GSTe2* mutations specific to East Kenya and South Africa (A,B) and common substitutions (Cys115Phe/Ser and Leu111Ser) together with west and central Africa specific Ile169Ser substitution. The residue at position 111 is methionine because we used PDB 2IMI structure of *An. gambiae* to show accurate ligand docking (66).**


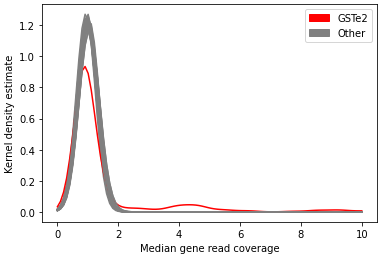


**Supplementary Figure 4. Median per-base read coverage across samples for GSTe2 and other genes. The coverage was normalised for each sample using median coverage across the genes for that sample. Two peaks are visible in GSTe2 at 4 and 9 median gene read coverage.**
